# Caffeine Induces Cell Death via Activation of Apoptotic Signal and Inactivation of Survival Signal in Human Osteoblasts

**DOI:** 10.3390/ijms9050698

**Published:** 2008-05-08

**Authors:** Pin-Zhen Lu, Ching-Yu Lai, Wen-Hsiung Chan

**Affiliations:** Department of Bioscience Technology and Center for Nanotechnology, Chung Yuan Christian University, Chung Li, Taiwan 32023

**Keywords:** caffeine, ROS, apoptosis, necrosis, osteoporosis

## Abstract

Caffeine consumption is a risk factor for osteoporosis, but the precise regulatory mechanisms are currently unknown. Here, we show that cell viability decreases in osteoblasts treated with caffeine in a dose-dependent manner. This cell death is attributed primarily to apoptosis and to a smaller extent, necrosis. Moreover, caffeine directly stimulates intracellular oxidative stress. Our data support caffeine-induced apoptosis in osteoblasts via a mitochondria-dependent pathway. The apoptotic biochemical changes were effectively prevented upon pretreatment with ROS scavengers, indicating that ROS plays a critical role as an upstream controller in the caffeine-induced apoptotic cascade. Additionally, p21-activated protein kinase 2 (PAK2) and c-Jun N-terminal kinase (JNK) were activated in caffeine-treated osteoblasts. Experiments further found that PAK2 activity is required for caffeine-induced JNK activation and apoptosis. Importantly, our data also show that caffeine triggers cell death via inactivation of the survival signal, including the ERK- and Akt-mediated anti-apoptotic pathways. Finally, exposure of rats to dietary water containing 10~20 μM caffeine led to bone mineral density loss. These results demonstrate for the first time that caffeine triggers apoptosis in osteoblasts via activation of mitochondria-dependent cell death signaling and inactivation of the survival signal, and causes bone mineral density loss *in vivo*.

## 1. Introduction

Caffeine and its related methyl xanthine products are widely distributed in plants, and people consume caffeine beverages every day. Common sources of caffeine include coffee, cocoa beans, cola nuts and tea leaves. Caffeine is probably the most commonly consumed pharmacologically active compound in the world, particularly in Europe and North America. Each cup of tea, coffee, cocoa and cola contains 70 mg, 192 mg, 5 mg and 40 mg of caffeine, respectively [1]. The caffeine level is estimated as 21.22 μM in the blood circulation of people imbibing 2427 mg caffeine for 36 weeks [1]. However, it is difficult to determine the exact physiological concentration of caffeine. The use of caffeine as a risk factor for bone loss is currently a subject of controversy. Earlier studies disclose a negative effect of caffeine on calcium economy and caffeine-induced urinary calcium loss by increased diuresis [2, 3]. In addition, caffeine possibly increases the risk of osteoporosis, as deduced from the decrease in bone mineral density (BMD) [4], increase in hip fracture risk [5], and negative effects on calcium retention [6, 7]. While several studies show that caffeine-containing beverage consumption is associated with reduced bone mass and increased fracture risk, the precise mechanisms by which caffeine induces osteoporosis remain to be elucidated.

The cytotoxicity of caffeine may be due to its ability to trigger apoptosis [8]. A number of cysteine proteases, known as caspases, play important roles in apoptosis [9, 10], similar to members of the Bcl-2 family [11], which regulate mitochondrial membrane potential changes and the release of cytochrome C by modulating the outer mitochondrial membrane permeability. Moreover, changes in protein kinase activity are observed during apoptosis in a variety of cell types [12], indicating that phosphorylation is involved in cell death regulation. In particular, c-Jun N-terminal kinase (JNK) acts as a key component that regulates entry into apoptosis in several cell types [13–15]. In addition to JNK, p21-activated kinase (PAK) is activated during apoptosis, and may also be involved in signaling events [16–20]. While the direct downstream substrates of PAKs are largely unknown, recent studies suggest that these proteins act as upstream regulators of JNK and p38 MAPK pathways [21, 22].

HSP90, a 90 kDa isoform of the HSP family proteins, acts in concert with other chaperones and partners to facilitate the maturation and folding of client proteins via formation of a HSP90/multi-chaperone complex [23]. Two HSP90 client proteins, Raf-1 and MAPK/ERK kinase (MEK), are components of the Ras/extracellular signal regulated kinase (ERK)-dependent pathway that is involved in both proliferation and anti-apoptosis [24]. The second most important anti-apoptotic pathway involves Akt/PKB, another HSP90 client protein [25, 26]. The protective effect of Akt against apoptosis could be attributed to the modulation of mitochondria-dependent bcl-2 proteins [27]. However, there are no documented reports on the effects of caffeine on ERK- or Akt/PKB-mediated survival signaling in osteoporosis.

To elucidate the precise regulatory mechanisms of caffeine-triggered cytotoxicity in osteoporosis, we examined the effects of caffeine on human osteoblasts. Based on the present results, we propose a model of caffeine-induced cell injury signaling in osteoblasts. *In vivo* experiments showed that caffeine consumption causes bone mineral density loss in an animal assay model, possibly as a result of cytotoxicity.

## 2. Results and Discussion

**Figure 1.**
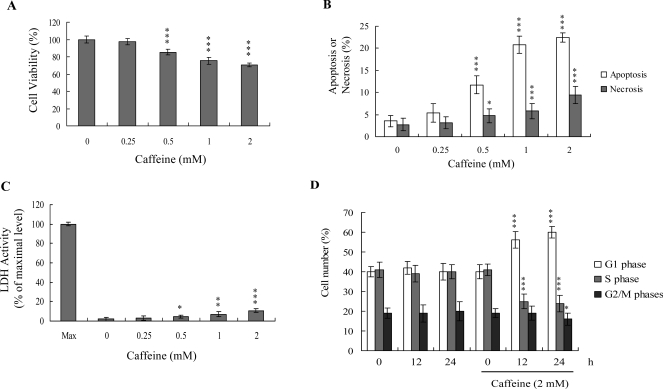
**Effects of caffeine on osteoblasts.** Osteoblasts were incubated with various concentrations of caffeine for 24 h. (A) Cell viability was determined using the MTT assay. (B) The percentages of apoptosis and necrosis were determined by propidium iodide and Hoechst 33342 staining. (C) Activity of LDH released in the culture medium of osteoblasts after treatment with various concentrations of caffeine for 24 h was measured. Data are expressed as a percentage of the maximal level (Max) of LDH activity determined after total cell lysis. (D) Osteoblasts were treated with or without 2 mM caffeine for various time periods as indicated. Cell cycle distribution of caffeine-treated osteoblasts was measured by flow cytometry analysis of propidium iodide-labeled cells. Values are presented as means ± SD of eight determinations. *P<0.05, **P<0.01 and ***P<0.001 versus the untreated control group.

Previous studies show that caffeine induces various cell responses, including cell death [28]. However, the effects of caffeine on osteoblasts and osteoporosis are currently unclear. The potential cytotoxicity of caffeine was examined by determining the viability of human osteoblasts treated with various doses of the compound, using the MTT assay. Osteoblasts were incubated in medium containing 0–2 mM caffeine for 24 h. The viability of treated osteoblasts was decreased by approximately 10–35% at concentrations higher than 0.5 mM caffeine in a dose-dependent manner (Figure 1A). We further investigated whether caffeine-induced cell death represents apoptosis or necrosis. The percentage of apoptotic cells increased significantly in cultures exposed to >0.5 mM caffeine, and the necrotic cell population simultaneously increased at higher concentrations (Figures 1B and C). These results indicate that treatment with caffeine triggers two cell death modes in osteoblasts, primarily apoptosis, and to a smaller extent, necrosis (Figures 1B and C). In addition, the DNA content of various cell cycle phases was determined by flow cytometry analysis of propidium iodide-labeled cells (Figure 1D). The decline in osteoblast survival ratio following treatment with caffeine was attributed to the simultaneous occurrence of G1 arrest, apoptosis and necrosis (Figures 1B and 1D).

**Figure 2.**
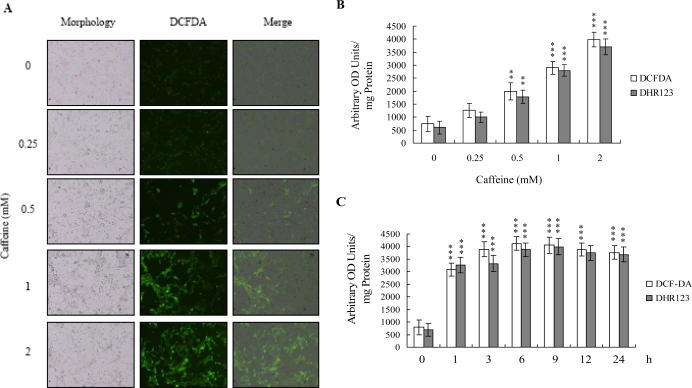
**Caffeine induces ROS generation in osteoblasts.** Osteoblasts were incubated with 20 μM DCF-DA or dihydrorhodamine 123 (DHR 123) for 1 h, and treated with various concentrations of caffeine for another 2 h. (A) Cells were observed using a fluorescence microscope at 100-fold magnification under fluorescence illumination. (B) ROS generation was assayed by DCF-DA or DHR 123 fluorescence, and expressed as absorbance/mg of protein. (C) Osteoblasts were treated with 2 mM caffeine for various time periods as indicated. ROS generation was assayed by DCF-DA or DHR 123 fluorescence. Values are presented as means ± SD of eight determinations. **P<0.01 and ***P<0.001 versus untreated control group.

There is no documented evidence to show that caffeine directly provokes oxidative stress in cells. However, several reports demonstrate that ROS are effective cell injury inducers, leading to apoptosis and necrosis [29]. Therefore, we examined whether ROS formation occurs in caffeine-treated osteoblasts by immunostaining analysis with DCF-DA as the detection reagent. As shown in Figure 2A, treatment with 0–2 mM caffeine for 24 h enhanced the intracellular ROS content in osteoblasts. ROS generation was additionally measured with DCF-DA and DHR-123 fluorescence dyes using the fluorescence ELISA reader (Figure 2B). In addition, ROS generation can detect when cells treatment with caffeine for more than 1 h (Figure 2C). To our knowledge, this is the first study to show that caffeine directly induces ROS generation in osteoblasts.

**Figure 3.**
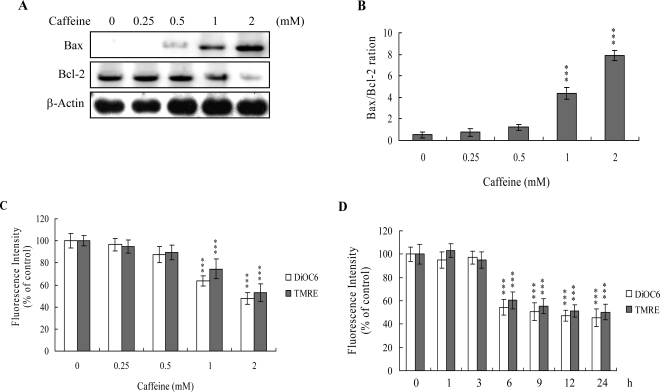
**Caffeine induces an increase in the ratio of Bax/Bcl-2 protein level and loss of MMP in osteoblasts.** Osteoblasts were incubated with various concentrations of caffeine for 24 h. (A) Extracts (40 μg) were analyzed by immunoblotting with anti-Bcl-2 or Bax antibodies. (B) Bax and Bcl-2 levels were analyzed using densitometry. (C) MMP changes were analyzed with 40 nM DiOC6(3) or 1 μM TMRE. (D) Osteoblasts were treated with 2 mM caffeine for various time periods as indicated. MMP changes were analyzed with 40 nM DiOC6(3) or 1 μM TMRE. Values are presented as means ± SD of eight determinations. **P<0.01 and ***P<0.001 versus untreated control group.

The protein expression ratio of Bax versus Bcl-2 is relevant to apoptosis. Specifically, a high Bax/Bcl-2 ratio is associated with a lower threshold of apoptosis, while a low ratio represents a higher apoptotic threshold [30, 31]. Here, we investigate whether caffeine induces apoptosis by modulating the Bax/Bcl-2 ratio, the major effectors of mitochondria-mediated apoptosis. Immunoblotting revealed that treatment of osteoblasts with more than 0.5 mM caffeine triggered an increase in Bax and decrease in Bcl-2 protein levels (Figure 3A). Densitometric analysis quantitatively revealed that caffeine-treated osteoblasts have a higher Bax/Bcl-2 ratio, favoring apoptosis (Figure 3B).

Bax and Bcl-2 regulate changes in the mitochondrial membrane potential (MMP) and permeability, which play important roles in apoptotic processes [32]. As mitochondrial membrane potential change is directly associated with apoptosis [33–35], we further investigated the effects of caffeine on this parameter. Treatment of osteoblasts with caffeine decreased DiOC6(3) and TMRE uptake into mitochondria, indicating that caffeine induces significant loss of mitochondrial membrane potential (Figure 3C). In addition, we also found that loss of MMP was detectable when cells treatment with caffeine for more than 6 h (Figure 3D). Our findings support the theory that caffeine triggers apoptosis via a mitochondria-dependent pathway.

**Figure 4.**
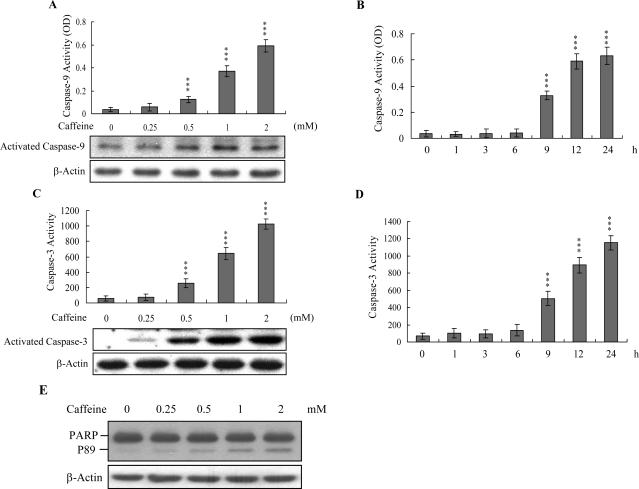
**Activation of caspase-9 and caspase-3 in caffeine-treated osteoblasts.** Osteoblasts were incubated with various concentrations of caffeine for 24 h. Cell extracts (40 μg) were immunoblotted using anti-caspase-9 (A), anti-caspase-3 (C), or anti-PARP (E) antibodies. P89 (89 kDa) represents the cleavage product of PARP. Caspase-9 activity was also assayed using the Colorimetric Caspase-9 Assay Kit (A; upper panel). The same cell extracts (60 μg) were analyzed for caspase-3 activity, using Z-DEVD-AFC as the substrate (C; upper panel). (B and D) Osteoblasts were treated with 2 mM caffeine for various time periods as indicated. Caspase-9 (B) and caspase-3 activities (D) were measured.

Caspases play important roles in apoptosis, and their activation is critical in biochemical changes for cell death analysis [9, 10]. For identifying the signaling pathways involved in caffeine-induced cell death, we used an immunoblotting assay to monitor the levels of caspases-9 and -3, which are activated during the apoptosis of multiple cell types triggered by various stimuli [36, 37]. Treatment of osteoblasts with caffeine stimulated caspase-9 and -3 activities (Figures 4A–4D). The activation of caspase-9 and caspase-3 were detectable when cells treatment with caffeine for more than 9 h (Figure 4B and 4D). Poly(ADP-ribose)polymerase (PARP) is a key participant in DNA base excision repair. Apoptosis is accompanied by early and unique cleavage of PARP. The formation of a 89 kDa cleavage fragment resulting from the specific PARP cleavage pattern is a hallmark of apoptosis. Here, we show that PARP, a specific caspase-3 substrate, is cleaved in caffeine-treated osteoblasts (Figure 4E). Accordingly, we propose that caffeine-triggered apoptosis occurs via caspase-9 and caspase-3 activation.

**Figure 5.**
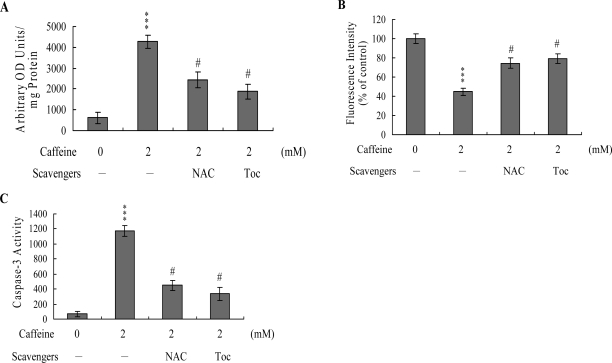
**Effects of ROS scavengers on caffeine-treated osteoblasts.** Osteoblasts were treated with or without the indicated concentrations of α-tocopherol (Toc; 300 μM) or N-acetyl cysteine (NAC; 500 μM) for 30 min, followed by incubation with caffeine (2 mM) for another 24 h. ROS generation (A), MMP changes (B), and caspase-3 activities (C) were measured. Values are presented as means ± SD of eight determinations. ***P<0.001 versus untreated control group. #*P*<0.001 versus “caffeine-treatment only” group.

To further clarify the role of ROS in caffeine-induced apoptosis, we assessed the effects of two commonly used ROS scavengers, N-acetyl cysteine (NAC) and α-tocopherol, on caffeine-treated osteoblasts. Pretreatment of cells with α-tocopherol (300 μM) or NAC (500 μM) attenuated caffeine-induced intracellular ROS generation, loss of MMP, and caspase-3 activation (Figures 5A, B and C). Based on the results, we propose that caffeine triggers ROS generation, which in turn activates mitochondria-dependent apoptotic processes in osteoblasts.

**Figure 6.**
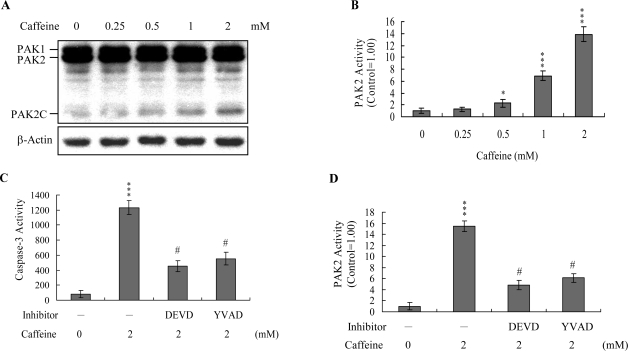
**Caffeine induces the cleavage/activation of PAK2.** Osteoblasts were incubated with various concentrations of caffeine for 24 h. (A) Cell extracts (60 μg) were immunoblotted with anti-αPAK (C19) antibody. (B) PAK2 was immunoprecipitated, and kinase activities were assayed using myelin basic protein as the substrate. (C and D) Osteoblasts were preincubated in the presence or absence of 300 μM Ac-DEVD-cho or Ac-YVAD-cmk for 1 h, and then incubated with 2 mM caffeine for another 24 h. Caspase-3 (C) and PAK2 (D) activities were measured. Values are presented as means ± SD of five determinations. *P<0.05 and ***P<0.001 versus the untreated control group. *#P*<0.001 versus caffeine treatment group.

Previous studies show that p21-activated protein kinase 2 (PAK2) is activated during cell death, and may be involved in apoptotic signaling events, possibly via caspase-3-directed proteolysis [18–20]. We investigated whether PAK2 cleavage/activation occurs in caffeine-treated osteoblasts. Immunoblotting and immunoprecipitation kinase activity assays revealed that caffeine induces PAK2 cleavage (Figure 6A) and activation (Figure 6B), respectively. Caspase-3 is activated during apoptosis of several cell types triggered by a variety of stimuli, including anti-Fas antibodies, TNF-α, and the chemotherapeutic agent, etoposide [36, 37]. To elucidate the relationship between caspase-3 activation and PAK2 during caffeine-induced apoptosis, we examined the effects of the specific tetrapeptidic caspase inhibitors, Ac-DEVD-cho and Ac-YVAD-cmk [18, 20], on caffeine-treated osteoblasts. Caffeine-induced activation of caspase-3 was markedly suppressed upon pretreatment with either of the inhibitors (Figure 6C). Pretreatment with caspase inhibitors additionally blocked caffeine-induced PAK2 activity (Figure 6D), indicating that caspase-3 functions upstream of PAK2 in the apoptotic pathway.

**Figure 7.**
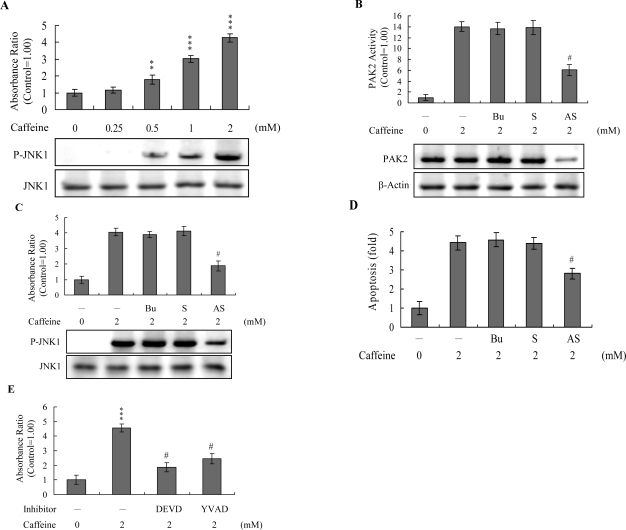
**Active PAK2 is required for JNK activation in caffeine-induced apoptosis.** (A) Osteoblasts were incubated with various concentrations of caffeine for 24 h. Cell extracts (60 μg) were prepared and immunoblotted with the anti-p-JNK antibody. JNK/AP-1 activity was additionally evaluated by ELISA analysis of phosphorylated c-Jun. Results are expressed with respect to untreated control values arbitrarily set to 1.00. (B-D) Osteoblasts were transfected with 70 μM PAK2 sense (S) or antisense (AS) oligonucleotides for 72 h, followed by 2 mM caffeine for another 24 h. PAK2 activity (B), JNK activity (C) and cell apoptosis (D) were measured. (E) Osteoblasts were preincubated in the presence or absence of 300 μM Ac-DEVD-cho or Ac-YVAD-cmk for 1 h, and then incubated with 2 mM caffeine for another 24 h. JNK/AP-1 activity was measured. **P<0.01 and ***P<0.001 versus untreated control group. #*P*<0.001 versus “caffeine-treatment only” group.

Activation of JNK is essential for apoptotic induction in some cell types [13, 14], and in earlier investigations, we demonstrate that UV irradiation-, methylglyoxal-, and high glucose-induced apoptosis are mediated by JNK [38–40]. Here, we use immunoblotting and ELISA assays to examine JNK activation during caffeine-induced apoptosis. JNK activity was stimulated in osteoblasts treated with caffeine (Figure 7A). To further determine the relationship between JNK and PAK2 and the functional role of PAK2 in caffeine-induced apoptosis, we incubated osteoblasts with antisense or sense oligonucleotides against PAK2 for 3 days, subjected cells to caffeine treatment, and analyzed cell extracts by immunoblotting with anti-PAK2 (N17) antibodies and immunoprecipitation. Pre-incubation of osteoblasts with an antisense oligonucleotide against PAK2 led to a significant decrease in PAK2 protein levels (by ~40%), compared to untreated controls, whereas the sense oligonucleotide had no such effect (Figure 7B). Similarly, cells treated with the antisense PAK2 oligonucleotide displayed PAK2 activation levels that were approximately 40% those of control cells (Figure 7B). These reductions in PAK2 protein expression and activation were associated with a significant decrease in caffeine-induced activation of JNK and apoptosis (Figures 7C and 7D). Furthermore, activation of JNK was markedly reduced in the presence of the caspase-3 inhibitors (Figure 7E). However, caspase inhibitors had no effects on ROS generation in caffeine-treated osteoblasts (data not shown). Our findings support the theory that ROS generation is an upstream regulator of sequential apoptotic biochemical changes, such as caspase activation. The data strongly suggest that PAK2 activation plays an important role in caffeine-induced JNK activation and apoptosis of osteoblasts.

Analysis of the results in Figure 7 reveals that the decreased ratios of PAK2 (~57%) and JNK activity (~54%) are higher than that of apoptosis inhibition (~36% decrease) in caffeine-treated cells. This finding indicates that other regulatory mechanisms are additionally involved in caffeine-induced apoptosis in osteoblasts.

**Figure 8.**
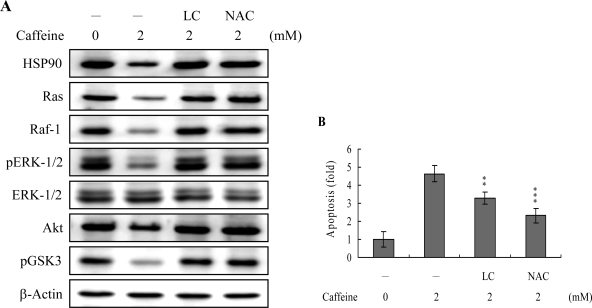
Effects of caffeine on HSP90 and components of survival signaling. Osteoblasts were incubated with or without lactacystin (LC; 10 μM) or NAC (500 μM) for 1 h, and treated with caffeine (2 mM) for another 24 h. (A) The protein levels of HSP 90, Raf-1, ERK-1/2 and Akt, and phosphorylation of ERK-1/2 and pGSK3 were evaluated. β-actin was used as the loading control. (B) Cell apoptosis was detected with the TUNEL assay. Data are representative of five independent experiments. **P<0.01 and ***P<0.001 versus “caffeine-treatment only” group.

Survival signaling processes protect against apoptosis induced by specific stimuli [41, 42]. Our group and others have demonstrated that some apoptotic stimuli inhibit the Ras→ERK survival signal pathway by decreasing the protein levels of various survival components [31, 42, 43]. Based on these results, we evaluated the effects of caffeine on the cell survival signal pathway, including activities of components critical for the Ras→ERK-dependent and PI3 Kinase→Akt survival signal pathways. Our results show that caffeine triggers a decrease in the protein levels of HSP90, Ras, Raf-1, and Akt, and inhibition of ERK and Akt activities (Figure 8A). In addition, inhibition of protein expression or activities of these survival signal components by caffeine could be effectively blocked by pretreatment with lactacystin, a specific proteasome inhibitor, and NAC, a potent ROS scavenger (Figure 8A). Moreover, lactacystin and NAC prevented caffeine-induced cell apoptosis, which correlates the blocking of survival signal components decreases (Figures 8A and B). These data imply that Ras, Raf-1, and Akt are degraded by ROS and proteasome-dependent pathways in caffeine-treated osteoblasts, subsequently reducing ERK and Akt activities for cell apoptosis.

**Figure 9.**
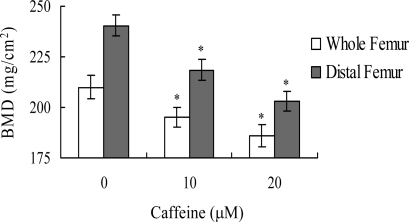
**Effects of caffeine intake on BMD of rats.** DXA was used to assess BMD in whole femurs and distal femurs of rats fed a controlled diet with or without caffeine (10 or 20 μM) in the drinking water for 8 months. Values are presented as means ± SD of five determinations. *P<0.05 versus “caffeine-free” group.

Lastly, we analyzed whether the apoptotic effect of caffeine on osteoblasts induces bone density injury *in vivo*. Wistar rats were fed a standard diet supplemented with or without caffeine (10 or 20 μM) in drinking water for 8 months. Initial body weights were similar among the control and caffeine-treated groups, and did not differ significantly at the end of the 8-month experimental period (data not shown). However, the bone mineral densities (BMD) of whole femurs and distal femurs from rats in the caffeine-treated groups were significantly lower than those in the control (caffeine-free) group (Figure 9). These results suggest that elevated caffeine levels, resulting from continuous long-term consumption of high caffeine-containing foods, negatively affect BMD, an important osteoporosis indicator.

Substantial amounts of caffeine are ingested by people drinking coffee, tea or caffeinated soft drinks. While the consumption of caffeine-containing beverages is associated with significantly increased osteoporosis risk, its precise role and regulatory mechanisms in bone loss and fracture risk are currently unclear [44]. Our current results provide evidence that the apoptotic and necrotic properties of caffeine contribute to bone loss and osteoporosis in osteoblasts. The majority of caffeine-treated cells die via apoptotic processes through ROS generation and a mitochondria-mediated pathway, while a minor proportion of cell death occurs through necrosis (Figures 1–3).

Oxidative stress is a stimulator of cell responses such as apoptosis [29, 45]. Importantly, caffeine directly induced oxidative stress in osteoblasts in our study (Figure 2). In addition, pretreatment with antioxidants effectively prevented ROS-induced apoptotic biochemical changes (Figure 4). Several reports show that ROS is an important upstream regulator of JNK and caspase activation during UV-, methylglyoxal-, and osmotic shock-induced apoptosis in various mammalian cells [38, 40, 46]. These results, coupled with our findings, strongly imply that oxidative injury plays a pivotal role in caffeine-induced apoptosis. However, the underlying mechanisms of caffeine-triggered ROS formation remain to be established. The effects of caffeine on target cells may depend, at least in part, on cell type and treatment protocol (i.e., treatment period and dosage).

Mitochondria act as important conduits for signals during programmed cell death, and loss of mitochondrial integrity can be promoted or inhibited by several key regulators of apoptosis [47, 48]. For instance, various cellular stress conditions, including heat shock, DNA damage and oxidative stress, result in caspase activation through cytochrome c release from the mitochondrial intermembrane space into the cytoplasm [48, 49]. We examined Bax/Bcl-2 protein expression and MMP changes, with a view to further elucidating the precise mechanisms of caffeine-induced apoptosis. Caffeine triggered an increase in Bax/Bcl-2 protein expression and loss of MMP in a dose-dependent manner (Figures 3A–3C). Moreover, caspases-9 and -3 were activated in the presence of caffeine (Figure 4). Interestingly, caffeine induced ROS generation and activation of caspase-9 and caspase-3 at concentrations higher than 0.5 mM, but had no effects on MMP changes or the Bax/Bcl-2 ratio at 0.5 mM (Figures 2, 3 and 4). These ambiguous results may be affected by the different assay sensitivities of various methods or assay kits. To elucidate the underlying reasons for these conflicting results, we further analyzed Bax and Bcl-2 mRNA levels by real-time PCR. Our analyses revealed that caffeine treatment of osteoblasts led to increased and decreased Bax and Bcl-2 expression levels at concentrations higher than 0.5 mM, respectively (data not shown). Thus, apoptotic biochemical changes, such as ROS generation, MMP changes, and activation of caspases appear to be correlated. In addition, inhibition of ROS generation by antioxidants was associated with a decrease in caffeine-triggered mitochondria-dependent apoptotic changes (Figure 5). Accordingly, we propose that ROS act as important regulators of mitochondria-dependent apoptotic processes promoted by caffeine.

Recently, coworkers of our laboratory and other researchers showed that PAK2 is a target substrate for caspases activated by various apoptotic stimuli [17–19, 50]. However, the functional role of the caspase-generated C-terminal active fragment of PAK2 remains obscure. Our group showed that decreases in PAK2 protein expression and activation are associated with significant inhibition of methylglyoxal-induced apoptosis in human osteoblasts [30], strongly suggesting that PAK2 plays an important role in apoptosis by methylglyoxal. Here, we demonstrate the PAK2 is an important upstream regulator of JNK activation in caffeine-directed apoptosis of osteoblasts (Figures 6 and 7). JNK evidently plays critical roles in apoptosis [14, 15]. Moreover, inhibition of caspase-3 blocks activation of PAK2 and JNK (Figures 6C, 6D, and 7E). Based on these results, we propose that caffeine triggers caspase-3 activation, which in turn induces cleavage/activation of PAK2 and sequential activation of JNK, consequently leading to apoptosis.

Heat-shock proteins (HSP) protect proteins against proteasome- and ubiquitin-dependent degradation [51, 52]. HSP90, the most abundant molecular chaperone protein in the intracellular system, is involved in maintaining the correct conformation of intracellular proteins and kinases, such as Raf-1 and Akt [53, 54], which regulate cell proliferation and survival. Here, we show that caffeine-induced apoptosis is associated with reduced expression of survival components, including Ras, Raf-1, and Akt. Moreover, inactivation of ERK-1, ERK-2 and Akt/PKB, and pretreatment with lactacystin, a proteasome inhibitor, may prevent these decreases in protein level or activity (Figure 8A). Caffeine suppresses HSP90 expression, thus promoting the degradation of client proteins (Figure 8A). Accordingly, we hypothesize that the caffeine-induced reduction of HSP90 stimulates Ras, Raf-1 and Akt targeting for degradation, leading to their downregulation and changes in the related signal pathways. In view of these findings, the following pathway is proposed: caffeine decreases HSP90 expression, leading to increased degradation of Ras, Raf-1 and Akt/PKB, with decreased Raf-1 levels resulting in ERK-1 and ERK-2 downregulation and subsequent cell apoptosis. ROS generation is additionally involved in caffeine-induced suppression of the levels of survival signal proteins (Figures 8A and B). Thus, it appears that ROS act as upstream negative regulators of Ras→ERK and PI3K→Akt/PKB survival signals in caffeine-induced apoptosis. However, the precise regulatory mechanisms require further investigation.

Lastly, given the ability of caffeine to induce apoptosis or cell injury in osteoblasts *in vitro*, we examined its effects on bone density in an animal assay model. Rats administered 10 or 20 μM caffeine in the drinking water for 8 months displayed significantly decreased bone mineral density (BMD), compared to untreated control rats, further signifying that caffeine consumption negatively affects bone density (Figure 9).

## 3. Conclusions

**Figure 10.**
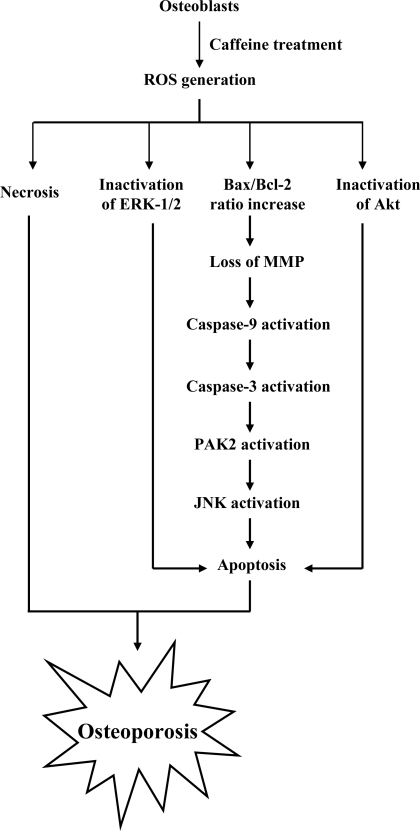
Summary of events occurring during caffeine-induced cytotoxicity in osteoblasts.

Recent study by our group found that the common signaling pathway in caffeine-stimulated cell injury occurs not only in the human osteoblast cell line, but also mouse osteoblast primary cultures and cell lines. The current results establish that caffeine-induced ROS trigger cell injury through apoptosis, necrosis and inactivation of survival signal components. In addition, a time-course study of various apoptotic biochemical changes indicates that ROS act as upstream regulators for MMP loss, which further triggers caspase activation for sequential apoptotic processes, such as activation of PAK2 and JNK. Based on these findings, we propose a model of caffeine-induced cell injury signaling in osteoblasts and possible effects on osteoporosis (specified in Figure 10). Our findings provide preliminary evidence that high consumption of caffeine is a significant risk factor in osteoporosis.

## 4. Experimental Section

### Chemicals

Caffeine, 2',7'-dichlorofluorescein diacetate (DCF-DA), propidium iodide, and Annexin V were purchased from Sigma (St. Louis, MO). Anti-phospho-JNK, Bax, caspase-3 and PAK2 antibodies were obtained from Santa Cruz Biotechnology (Santa Cruz, CA). Anti-poly (ADP-ribose) polymerase (PARP), Bcl-2, and caspase-9 antibodies were from Cell Signaling Technology (Beverly, MA). MTT [3-(4, 5-dimethylthiazol-2-yl)-2, 5-diphenyltetrazolium bromide] and the cell death detection ELISA^plus^ kit were acquired from Roche Applied Science (Germany). The BCA protein assay kit was purchased from Pierce Biotechnology (Rockford, IL, USA).

### Cell culture and caffeine treatment

The human osteoblast cell line, hFOB 1.19, was cultured according to the standard procedures of American Type Culture Collection (ATCC). Cells were seeded in 1:1 mixture of Ham's F12 medium and Dulbecco's modified Eagle's medium (DMEM/F12) without phenol red containing 2.4 mM L-glutamine, 0.3 mg/ml G418, and 10% fetal bovine serum media. Cultures were maintained at 34°C with a gas mixture of 5% CO2/95% air. For caffeine treatment, cells (~5–6 × 10^6^) were plated on 60 mm culture dishes, and incubated in medium containing various concentrations of caffeine for 24 h. Next, cells were washed twice with ice-cold PBS, and lysed in 600 μl of lysis solution (20 mM Tris-HCl, pH 7.4, 1 mM EDTA, 1 mM EGTA, 1% Triton X-100, 1 mM benzamidine, 1 mM phenylmethylsulfonyl fluoride, 50 mM NaF, 20 mM sodium pyrophosphate and 1 mM sodium orthovanadate) on ice for 10 min. Cell lysates were collected, sonicated on ice for 3 × 10 sec, and centrifuged at 15,000 × g for 20 min at 4°C. The resulting supernatant fractions were used as cell extracts.

### MTT assay

The MTT (3-[4,5-dimethylthiazol-2-yl]-2,5-diphenyltetrazolium bromide) test is a colorimetric assay that measures the percentage of cell survival. Caffeine-treated cells were treated with 100 μl of 0.45g/l MTT solution per dish, and incubated at 37°C for 60 min. This was followed by treatment with 100 μl of 20% SDS in DMF:H_2_O (1:1), and overnight incubation at 37°C. Spectrophotometric data were measured using an ELISA reader at a wavelength of 570 nm.

### Assessment of necrosis and apoptosis

Oligonucleosomal DNA fragmentation in apoptotic cells was measured using the Cell Death Detection ELISA^plus^ kit, according to the manufacturer's protocol (Roche Molecular Biochemicals, Mannheim, Germany). Cells (1×10^5^) were treated with or without the indicated concentrations of caffeine at 37°C for 24 h, and spectrophotometric data obtained with an ELISA reader at 405 nm. Necrosis and apoptosis were further assayed by staining with propidium iodide and Hoechst 33342. Specifically, cells were incubated with propidium iodide (1 μg/ml) and Hoechst 33342 (2 μg/ml) at room temperature for 10 min. Under a fluorescence microscope, propidium iodide-negative cells (having intact plasma membranes that exclude the dye) displaying condensed/fragmented nuclei stained with Hoechst 33342 were identified as apoptotic, whereas propidium iodide-positive cells were necrotic. In each experiment, 8–10 independent fields (~600–1000 nuclei in total) were counted per condition, and the cell percentages calculated. Necrosis was additionally assayed by measurement of lactate dehydrogenase (LDH) activity in the culture medium [55]. Cells were cultured in 96-well microtiter plates (100 μl medium/well), and absorption values at 490 nm were determined with an ELISA reader, according to the manufacturer's instructions (Promega, Madison, WI). Blanks consisted of samples containing test substances and cell-free medium.

### ROS assay

ROS levels were measured in arbitrary units using 2',7'-dichlorofluorescein diacetate (DCF-DA) and dihydrorhodamine 123 (DHR 123) dye. Cells (1.0×10^6^) were incubated in 50 μl PBS containing 20 μM DCF-DA or DHR-123 for 1 h at 37°C, and the relative ROS units determined using a fluorescence ELISA reader (excitation at 485 nm; emission at 530 nm). An aliquot of the cell suspension was lysed, and the protein concentration estimated. Results are expressed as arbitrary absorbance units/mg protein.

### Immunoblots

Immunoblotting was performed essentially as described in a previous report by our group [46]. Briefly, proteins were resolved by SDS-PAGE, transferred to PVDF membranes, and immunoblotted with anti-Bax, anti-Bcl-2, anti-caspase-3, anti-caspase-9, anti-poly (ADP-ribose) polymerase (PARP), anti-PAK (c-19), anti-JNK and anti-phospho JNK antibodies (0.25 μg/ml). Proteins of interest were detected with secondary alkaline phosphatase-conjugated goat anti-rabbit or anti-mouse IgG antibodies, and visualized using the CDP-Star^™^ chemiluminescent substrate, according to the manufacturer's protocol.

### Detection of mitochondrial membrane potential (MMP)

Osteoblasts were dispensed into 96-well plates, grown for 24 h, and incubated with various concentrations of caffeine for 24 h. Fluorescent dyes, DiOC6(3) (20 nM) or TMRE (0.1 μM), were added to each well, incubated for 15 min, and fluorescence measured with a plate spectrofluorometer [excitation: 485 nm (DiOC6(3)) or 535 nm (TMRE); emission: 535 nm (DiOC6(3)) or 590 nm (TMRE)].

### Caspase activity assays

Caspase-9 activity was determined using the Colorimetric Caspase-9 Assay Kit (Calbiochem, CA). Caspase-3 activity was measured using the Z-DEVD-AFC fluorogenic substrate, as described previously [30, 56].

### Immunoprecipitation and PAK2 activity assays

Prior to immunoprecipitation, cell extracts were diluted with cell lysis solution to a protein concentration of 1.0 mg/ml. For immunoprecipitation of the C-terminal catalytic fragment of PAK2, 0.5 ml of cell extracts was incubated with 10 μl of anti-PAK2 (C15) antibody (200 μg/ml) at 4°C for 1.5 h, and further incubated with 40 μl of Protein A-Sepharose CL-4B (30%, v/v) for 1.5 h with shaking. Immunoprecipitates were collected by centrifugation, washed three times with 1 ml of Solution A (20 mM Tris/HCl, pH 7.0, and 0.5 mM DTT) containing 0.5 M NaCl, and resuspended in 40 μl of Solution A. For measurement of PAK2 activity, immunoprecipitates were incubated in a 50 μl mixture containing 20 mM Tris/HCl, pH 7.0, 0.5 mM DTT, 0.2 mM [γ-p^32^]ATP, 20 mM MgCl_2_ and 0.1 mg/ml myelin basic protein at room temperature for 10 min with shaking. For determination of ^32^P incorporation into myelin basic protein, 20 μl of each reaction mixture was spotted onto Whatman P81 paper (1 × 2 cm), washed with 75 mM phosphoric acid and processed, as described previously [57].

### JNK activity assay

JNK activity, measured by the presence of phosphorylated c-Jun protein, was analyzed using the AP-1/c-Jun ELISA kit, according to the manufacturer's protocol (Active Motif, Carlsbad, CA). Briefly, AP-1 heterodimeric complexes in cellular nuclear extracts were collected by binding to the consensus 5'-TGA(C/G)TCA-3' oligonucleotide coated on a 96-well plate. Phospho-c-Jun was assayed using a specific primary antibody and secondary horseradish peroxidase-conjugated antibody in a colorimetric reaction.

### Inhibition of PAK2 by antisense oligonucleotides

PAK2 sense (5'-ATC ATG TCT GAT AAC GGA GAA) and antisense (5'-TTC TCC GTT ATC AGA CAT GAT) oligonucleotides (representing amino acids -1 to +7 of human PAK2) were obtained from Life Technologies (Grand Island, NY). Oligonucleotides were synthesized under phosphorothioate-modified conditions, purified by HPLC, and dissolved in 30 mM HEPES buffer, pH 7.0. For transfection, cells grown in 60 mm culture dishes were incubated at 37°C in 1 ml of Opti-MEM I medium (modified Eagle's minimum essential medium buffered with HEPES and sodium bicarbonate) containing lipofectAMINE4 (12 μg) and oligonucleotides (70 μM) for 72 h (all reagents from Life Technologies, Grand Island, NY). Cells were treated with 2 mM caffeine, and extracts were analyzed as described above.

### AKT kinase assay

Cell extracts were incubated and immunoprecipitated with 10 μl of anti-Akt monoclonal antibody (200 μg/ml). Details of the procedure are described in Section 2.9. Briefly, immunoprecipitates were incubated with 1 mg GSK-3 fusion protein (Cell Signaling Technology) in the presence of 200 mM ATP and reaction buffer (25 mM TRIS, pH 7.5, 5 mM β glycerophosphate, 2 mM dithiothreitol, 0.1 mM sodium orthovanadate, and 10 mM MgCl_2_) at 30°C for 30 min. The reaction mixture was analyzed with 12.5% SDS-PAGE, and immunoblotted with anti-phospho-GSK-3α/β (Ser21/9) rabbit polyclonal antibody.

### Animals, diets, and bone mineral density measurement

The effects of caffeine intake on bone mass were analyzed in 18 week-old female Wistar rats. Rats were randomly divided into three diet groups of 10 animals each. Group one was administered a standard diet, Group two fed a standard diet supplemented with 10 μM caffeine in the drinking water, and Group Three given a standard diet supplemented with 20 μM caffeine in the drinking water. Rats were exposed to caffeine-containing or control drinking water for 8 months, and the right femur of each rat was analyzed for bone mineral density (BMD) using dual energy X-ray absorptiometry (DXA). *In vivo* reproducibility was evaluated by measuring the coefficient of variation over five repeat experiments, and rats repositioned between readings. The same researcher conducted all DXA scans and analyses.

### Statistics

Data were analyzed using one-way ANOVA. Differences were evaluated using the Student's t-test and analysis of variance. Data are presented as means ± SD. *P* < 0.05 was defined as a significant difference.
